# Construction and Implications of Nomogram for Predicting Sustained Glycemic Remission After Short‐Term Intensive Insulin Therapy in Newly Diagnosed Type 2 Diabetes

**DOI:** 10.1111/1753-0407.70135

**Published:** 2025-08-01

**Authors:** Lijuan Xu, Liehua Liu, Zhiwei Xie, Zhimin Huang, Hai Li, Juan Liu, Xiaoying He, Wanping Deng, Yanbing Li

**Affiliations:** ^1^ Department of Endocrinology The First Affiliated Hospital, Sun Yat‐Sen University Guangzhou China; ^2^ Peking Union Medical College Hospital, Chinese Academy of Medical Sciences & Peking Union Medical College Beijing China

**Keywords:** glycemic remission, intensive insulin therapy, predictive nomogram

## Abstract

**Background:**

Early short‐term intensive insulin therapy may induce sustained glycemic remission in type 2 diabetes. However, patients' responses vary greatly. Exploring key factors to improve glycemic control and predict the probability of remission precisely is meaningful for future treatment.

**Methods:**

Patients with newly diagnosed type 2 diabetes receiving 2–3 weeks of intensive insulin therapy were followed up for at least 1 year in three randomized clinical trials. Data of theirs were extracted with the same inclusion criteria and divided into training and validation sets. A nomogram was constructed in the training set and tested in the internal validation set.

**Results:**

Among 302 patients with transient intensive insulin therapy, 162 (53.64%) patients achieved the 1‐year glycemic remission. Severe hyperglycemia at baseline did not impede future remission, as the remission rate was 60.70% in those with HbA1c greater than 13%. Three parameters were identified as predictive factors in the nomogram: fasting glucose after short‐term insulin treatment, mean glucose during insulin therapy, and postprandial glucose/fasting glucose ratio at baseline. The odds ratios were 0.06 (95% CI, 0.02–0.25), 0.41 (0.18–0.93) and 6.55 (1.39–30.89), respectively. Incorporating these factors, the nomogram achieved an accuracy of 81.50%.

**Conclusion:**

Short‐term intensive insulin therapy assists patients with newly diagnosed type 2 diabetes and significant hyperglycemia to achieve glycemic remission. A rigorous control of glucose during insulin treatment favors future remission. We construct a nomogram to predict sustained glycemic remission, which may help determine whether subsequent medication is needed and thus reduce both overtreatment and therapeutic inertia.

## Graphical abstract

Early short‐term intensive insulin therapy (IIT) may induce glycemic remission in type 2 diabetes. Patients with better fasting plasma glucose (FPG) after IIT, lower mean glucose (MG) during IIT and higher postprandial‐to‐fasting glucose ratio (PPG/FPG) at baseline are more likely to achieve sustained glycemic remission. A nomogram integrating these parameters effecitvely stratifies patients by their probability of maintaining long‐term glycemic remission.

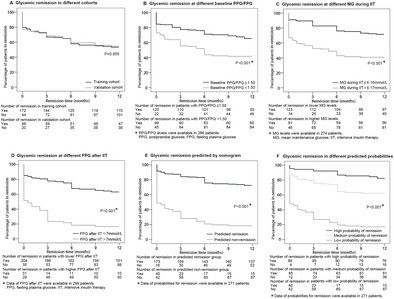

**Summary**

- Short‐term intensive insulin therapy can induce sustained glycemic remission, and severe hyperglycemia at baseline does not impede remission.
- This nomogram underscores the importance of stringent glucose control during intensive insulin therapy as the sole modifiable factor for achieving long‐term glycemic remission.
- The nomogram facilitates patient counseling and individualizes sequential treatment.

## 1 Introduction

Despite advances in treatment and glucose monitoring systems, patients with type 2 diabetes mellitus (T2DM) cannot be fully cured, since their islet function is believed to deteriorate inexorably [1]. However, as evidence from several weight‐loss diet studies and bariatric surgery trials accumulates, the concept that T2DM is a partially reversible syndrome is progressively recognized and confirmed [2, 3, 4, 5, 6, 7]. A recent consensus report redefined the term diabetes remission and noted that some patients with diabetes may return to normal glucose levels after medication withdrawal [8]. Furthermore, in our established series of studies, patients receiving early continuous subcutaneous insulin infusion (CSII) were able to improve β‐cell function and achieve a sustained drug‐free glycemic remission or simplify the subsequent treatment strategy [9, 10, 11, 12, 13, 14]. These findings challenge the traditional concept that progressive β‐cell failure is irreversible, highlight the feasibility of the intense‐simplified strategy in newly diagnosed T2DM patients with severe hyperglycemia, and emphasize the benefits of short‐term intensive insulin therapy [15, 16, 17].

In spite of these promising results, only half of patients responded well to the short‐term intensive insulin therapy (IIT). For the non‐responsive half, predicting the outcome and planning the subsequent treatment is necessary for sustained glucose control. Several predisposing factors have been found to correlate with long‐term glucose remission, such as red blood cell distribution width (RDW) at baseline [18], mean maintenance glucose (MG) during CSII [10], mild hypoglycemia episodes during CSII [19], and fasting plasma glucose (FPG) after short‐term CSII treatment [20]. However, these factors only imply that patients are more likely or unlikely to achieve remission. For example, according to our previous research, we can conclude that patients in the middle and lower tertiles of MG during CSII treatment have a higher 1‐year remission rate [10]. But the specific and accurate probability of remission is uncertain based on MG for a given patient. Therefore, the development of a quantitative tool to predict individualized long‐term glycemic control is essential.

Nomogram, a pictorial representation of a complex formula, is used to estimate prognoses in medicine [21, 22, 23]. To construct a nomogram, all of the determinant variables are listed separately, with corresponding scores assigned to the given magnitude, and the cumulative score of all variables is matched to the scale of prognostic probability. By graphically displaying the effect of each predictor, a more tangible interpretation can be provided. Due to its ability to derive prognostic probabilities quickly and intuitively, a nomogram will be useful for predicting the sustained glycemic remission probabilities and formulating sequential treatment strategies.

Hence, we comprehensively analyzed the essential features that might influence the sustained glycemic remission and conducted a nomogram to predict the glycemic remission in newly diagnosed T2DM after short‐term IIT.

## 2 Methods

### 2.1 Patients

The study protocols conformed to the provisions of the Declaration of Helsinki and were approved by the Research Ethics Committee of the First Affiliated Hospital, Sun Yat‐sen University. Data from three randomized clinical trials (NCT00147836, NCT00948324, and NCT01471808) conducted from 2004 to 2019 was re‐screened for eligibility based on the same inclusion and exclusion criteria [9, 24, 25]. All patients signed a written informed consent during those three trials. NCT00147836 was originally designed to compare the effect of CSII with other interventions (oral hypoglycemic agents or multiple daily injections) on long‐term glycemic control. A total of 331 patients completed the follow‐up, and the results demonstrated that early IIT had favorable outcomes on recovery and maintenance of β‐cell function and protracted glycemic remission. NCT00948324 was conducted to explore the different impacts on long‐term glycemic control in 400 patients with CSII alone or CSII combined with metformin, rosiglitazone, or α‐lipoic acid. It revealed that short‐term CSII in combination with rosiglitazone or metformin was superior to CSII alone in the third month, but similar to CSII alone at the 12th month point. NCT01471808 was carried out in 264 patients to evaluate the effects of CSII alone and CSII plus oral hypoglycemic agents (sitagliptin, or pioglitazone and metformin) on sustained glucose control. It showed that the remission rates of patients with CSII alone were the same as those receiving concurrent treatment with CSII and oral medications. To formulate the nomogram, only data of those who received CSII therapy alone in the three trials were analyzed.

Diabetes was diagnosed and classified according to the 1999 World Health Organization (WHO) criteria [26]. T2DM included the common major form of diabetes which resulted from insulin resistance or defects in insulin secretion. If it was challenging to differentiate between type 1 and type 2 diabetes in a patient, islet autoantibody testing would be conducted for a definitive diagnosis. As mentioned above, the unified inclusion and exclusion criteria were set for this study. Namely, patients with newly diagnosed T2DM, who had not used any hypoglycemic medications, aged 25–65 years old, with FPG levels of 7.0–16.7 mmol/L and body mass indexes (BMI) of 21–35 kg/m^2^ were included. Those who had infections, severe diabetic complications, or lost follow‐up in the original trials were excluded.

### 2.2 Study Design

CSII was provided to normalize the glucose and then maintained for 2 weeks after the glycemic target was reached. The glucose levels targeted less than 6.1 mmol/L for FPG and less than 8.0 mmol/L for 2‐h postprandial glucose (2 h PPG) in NCT00147836, 4.4–6.0 mmol/L for FPG and 4.4–8.0 mmol/L for 2 h PPG in NCT00948324 and NCT01471808. After insulin treatment, patients were followed up every 3 months without using any anti‐diabetic drugs until hyperglycemia recurred.

During hospitalization, in accordance with the Chinese Guidelines for the Prevention and Treatment of Type 2 Diabetes, the energy intake for patients was calculated based on a standard of 25–30 kcal per kilogram of their ideal body weight per day, and adjusted according to the patients' height, weight, age, and activity levels. Carbohydrates, protein, and fat accounted for 50%–60%, 10%–15%, and 20%–30% of total calories, respectively. Patients were encouraged to take a 1‐h post‐meal walk after each dinner. Life‐style modifications were suggested to be maintained after discharge. During the follow‐up phase, patients were required to recall their diet and exercise, and then we evaluated and provided tailored guidance for their subsequent life‐style modification.

Parameters including gender, age, levels of glucose, glycated hemoglobin A1c (HbA1c), BMI, blood pressure, lipids, RDW, fasting insulin, bilirubin, and creatinine at baseline, glucose levels and insulin dosage during the CSII treatment, as well as glucose, lipids, creatinine, and insulin levels after the cessation of CSII were compared. Those that exhibited statistically significant differences between the remission and the non‐remission group were regarded as potential predictors. These potential predictors were integrated into the logistic regression analysis to identify the independent predictors and develop a graphically intuitive nomogram.

Thus, clinicians can estimate the individualized probability of glycemic remission for patients undergoing early CSII therapy. Sustained glycemic remission was defined in this and our previous studies as FPG < 7.0 mmol/L, 2 h PPG < 10.0 mmol/L, and no need for antidiabetic agents for at least 1 year in the original trials. If glucose levels exceeded these values, hyperglycemic relapse was considered, and subsequent treatment was recommended depending on individual conditions and glucose levels [27].

## 3 Statistical Methods

All data were analyzed using SPSS 22.0 software packages (IBM, USA) and R statistics, version 4.0.0 (http://www.r‐project.org/). Normally distributed variables, expressed as the mean ± standard deviation (SD), were compared using *t*‐tests. Non‐normally distributed variables were expressed as the median (interquartile range) and analyzed via the rank‐sum test. Significant differences between the two groups were evaluated using the logistic regression model. Receiver operating characteristic (ROC) curves were constructed to determine the sensitivity and specificity. The cut‐off value of probability for one‐year glycemic remission was determined as the value with the highest Youden index score. The Box‐Tidwell method was used to determine the presence of a linear relationship between the log transforming value and remission possibility.

The data were randomly divided into the training or validation set. The training set comprised 70% of the total patient population, whereas the validation set represented 30%. The nomogram was formulated in the training set and tested in the validation one based on the results of logistic regression analysis using the R statistics. Each significant regression coefficient in the logistic regression was assigned 0–100 points in the nomogram. All points were added across the variables to determine the total points, which corresponded to the predicted probability. The predictive effect of the nomogram was evaluated by ROC curve analysis, a calibration plot, and decision curve analysis (DCA). The calibration plot and the concordance indexes, assessed by the Hosmer–Lemeshow test, were used to estimate the consistency between the predicted and observed glycemic remission status and to evaluate the predictive power of the nomogram. DCA was conducted to evaluate the predictive effect by quantifying the net benefit at different threshold probabilities. In this study, a two‐sided *p* value < 0.05 was considered statistically significant.

## 4 Results

### 4.1 General Conditions in the Remission and Non‐Remission Groups

**FIGURE 1:**
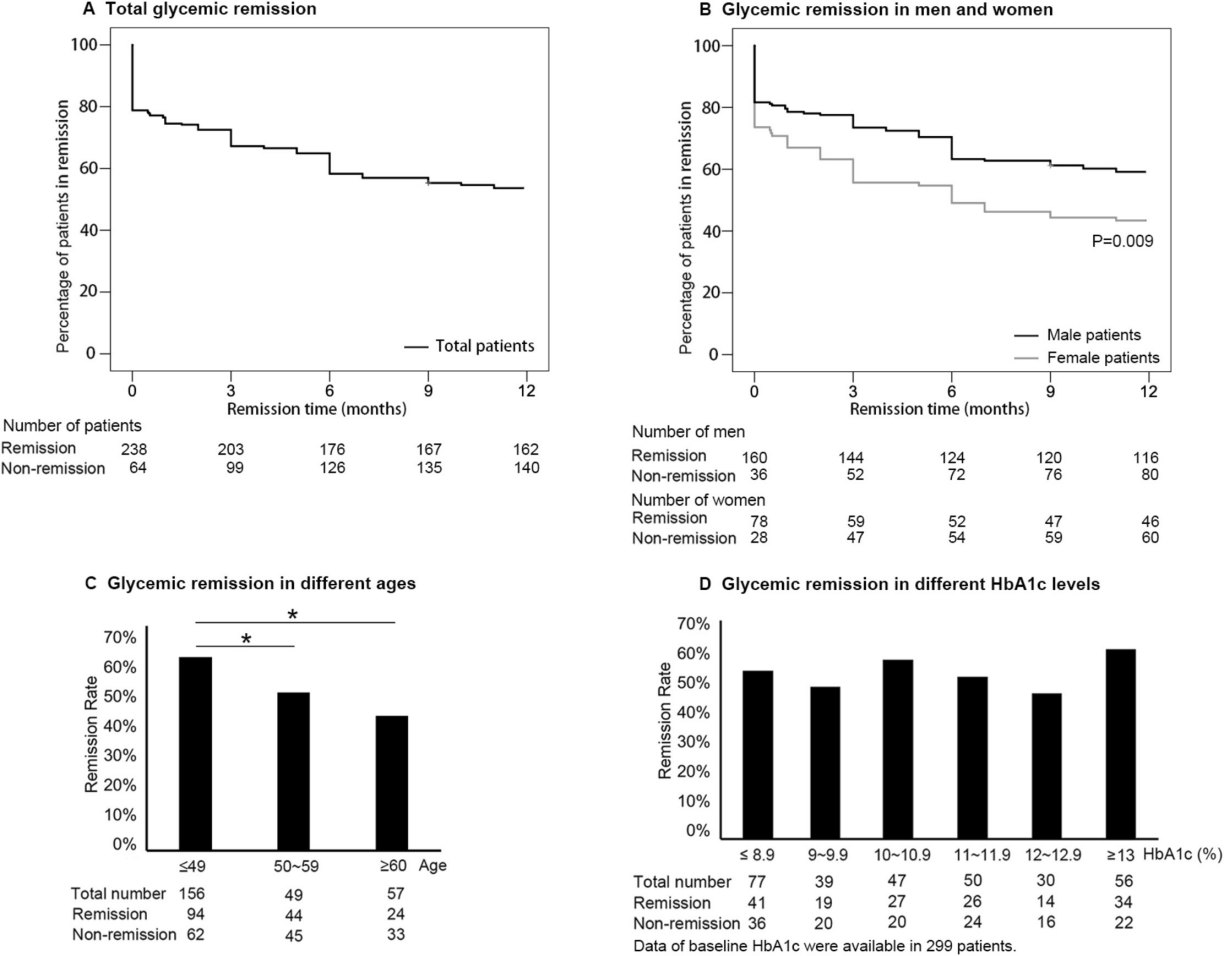
Kaplan–Meier survival curves and the Log‐Rank Test of glycemic remission rates in men and women, at different ages and baseline HbA1c levels. (A) The remission rate of total patients. (B) The remission rate in men was higher than that in women (*p* = 0.009). (C) The remission rates decreased as the ages of patients increased. (D) The remission rates of patients were consistent across different baseline HbA1c subgroups (*p* = 0.790). Those with HbA1c > 13% (118.58 mmol/mol) had a high remission rate of 60.70%. Three of the patients lacked baseline HbA1c records. *p < 0.05.

We collected data from 326 patients, aged 25–65 years old, with only CSII and without combined or subsequent therapies of oral anti‐diabetic medications, including 119 patients from NCT00147836, 98 patients from NCT00948324, and 109 patients from NCT01471808 (ClinicalTrials.gov). Five patients with infections, four patients lacking crucial data, and 15 patients lost to follow‐up were excluded. A total of 302 patients were included. As a result, the proportion of patients in drug‐free remission was 67.55% (204 of 302 patients) at 3 months of follow‐up, 58.61% (177 patients) at 6 months, 55.30% (167 patients) at 9 months, and 53.64% (162 patients) at 12 months (Figure 1A). The remission rates during the one‐year follow‐up in different subgroups were shown in Figure 1B–D. Predictably, the one‐year remission rates declined with age (*p* < 0.05). Surprisingly, however, patients with baseline HbA1c greater than 13% (118.58 mmol/mol) had a remission rate of up to 60.70%.

Although after short‐term CSII treatment, both the remission group and non‐remission group had significant improvements in glucose and β‐cell function from baseline, several differences were found between the two groups. Patients in the remission group were slightly younger and more male than female, with a bigger BMI and higher PPG‐to‐FPG ratio (PPG/FPG) at baseline, lower MG, and higher mild hypoglycemia incidence during CSII, better glucose control, and greater improvement in β‐cell function after short‐term CSII. The clinical characteristics of patients at baseline and during and right after CSII were shown in Table 1.

**TABLE 1:** The clinical characteristics of patients before, during and after short‐term CSII therapy.

	Total (*n* = 302)	Remission group (*n* = 162)	Non‐remission group (*n* = 140)	*p*
**Parameters at baseline**				
Age (years)	49.17 ± 10.48	47.87 ± 10.00	50.67 ± 10.85	0.020*
Gender (F/M)	106/196	46/116	60/80	0.009*
BMI (Kg/m^2^)	25.14 ± 3.17	25.51 ± 3.34	24.73 ± 2.94	0.033*
HbA1c (%)	10.69 ± 2.31	10.69 ± 2.35	10.69 ± 2.27	0.983
(mmol/mol)	93.32 ± 25.21	93.29 ± 25.65	93.35 ± 24.79	
FPG (mmol/L)	12.23 ± 3.42	11.85 ± 3.40	12.85 ± 3.84	0.017*
PPG (mmol/L)	18.32 ± 6.03	18.74 ± 6.40	17.83 ± 5.55	0.203
PPG/FPG	1.52 ± 0.38	1.61 ± 0.39	1.41 ± 0.33	< 0.001*
TG (mmol/L)	2.17 ± 2.13	2.19 ± 1.79	2.16 ± 2.48	0.893
HDL‐C (mmol/L)	1.14 ± 0.29	1.11 ± 0.29	1.17 ± 0.27	0.010
LDL‐C (mmol/L)	3.70 ± 1.11	3.66 ± 1.14	3.75 ± 1.09	0.460
Creatinine (μmol/L)	67.34 ± 18.34	69.02 ± 17.63	65.34 ± 19.02	0.086
ALT (U/L)	28.66 ± 16.40	30.19 ± 17.42	26.91 ± 15.04	0.093
AST (U/L)	32.95 ± 24.26	33.84 ± 21.87	31.95 ± 26.76	0.514
TBIL (μmol/L)	15.84 ± 5.87	15.52 ± 5.26	16.21 ± 6.51	0.353
RDW (fL)	39.31 ± 2.95	38.90 ± 3.06	39.77 ± 2.76	0.028*
HsCRP (mg/L)	1.63 (2.18)	1.76 (2.25)	1.41 (2.01)	0.179
AIR (pmol/L*10 min)	−105.01 (234.20)	−110.83 (259.78)	−95.99 (216.81)	0.614
HOMA‐B	21.90 (24.91)	22.81 (28.95)	20.47 (23.03)	0.088
HOMA‐IR	4.25 (4.10)	4.10 (4.56)	4.53 (4.75)	0.433
**Parameters during 2–3 weeks of CSII**				
Mean insulin dose (IU/day)	39.06 ± 12.54	38.81 ± 13.87	39.33 ± 10.87	0.722
MG (mmol/L)	6.31 ± 0.80	6.08 ± 0.59	6.60 ± 0.94	< 0.001*
Hypoglycemia episodes/day	0.29 (0.50)	0.36 (0.45)	0.21 (0.43)	0.017 *
Euglycemia obtained time				
1st–3rd day	138 (45.70%)	85 (51.52%)	53 (38.69%)	0.030*
4th–7th day	116 (38.41%)	61 (36.97%)	55 (40.14%)	
> 8th day	48 (15.89%)	19 (11.51%)	29 (21.17%)	
**Parameters after 2–3 weeks of CSII**				
HbA1c (%)	9.07 ± 1.80	9.00 ± 1.86	9.16 ± 1.73	0.455
(mmol/mol)	75.66 ± 19.70	74.87 ± 20.34	76.60 ± 18.93	
FPG (mmol/L)	6.29 ± 1.19	5.96 ± 0.86	6.68 ± 1.39	< 0.001*
PPG (mmol/L)	9.03 ± 3.17	8.42 ± 2.96	9.79 ± 3.28	< 0.001*
TG (mmol/L)	1.33 ± 0.57	1.28 ± 0.60	1.39 ± 0.54	0.084
HDL‐C (mmol/L)	1.23 ± 0.31	1.22 ± 0.30	1.24 ± 0.32	0.529
LDL‐C (mmol/L)	3.34 ± 1.00	3.35 ± 1.04	3.33 ± 0.95	0.843
Creatinine (μmol/L)	69.77 ± 15.32	71.70 ± 14.47	66.96 ± 16.13	0.019*
HsCRP (mg/L)	1.29 (2.15)	1.29 (2.49)	1.30(1.80)	0.833
AIR (pmol/L*10 min)	423.50 (681.65)	492.13 (785.38)	329.63 (512.20)	0.003*
HOMA‐B	62.58 (60.52)	63.72 (60.98)	57.99 (64.63)	0.061
HOMA‐IR	2.11 (2.15)	2.06 (1.83)	2.36 (2.33)	0.050

*Note:* Data are expressed as mean ± SD or median (interquartile range).

Abbreviations: AIR: acute insulin response; ALT: alanine transaminase; AST: aspartate aminotransferase; BMI: body mass index; Cr: creatinine; FPG: fasting plasma glucose; HbA1c: glycated hemoglobin A1c; HDL‐C: high density lipoprotein cholesterol; HOMA‐B: homeostasis model assessment of β‐cell function; HOMA‐IR: homeostasis model assessment of insulin resistance; HsCRP: high sensitivity C reactive protein; LDL‐C: low density lipoprotein cholesterol; MG: mean maintaining glucose after normoglycemia; PPG: postprandial plasma glucose; RDW: red cell distribution width; TBIL: total bilirubin; TG: triglyceride.

* *p* < 0.05 compared between the remission and non‐remission groups.

### 4.2 Assumption of Linearity in Logistic Regression Models

All parameters except FPG and PPG after CSII therapy were linearly correlated with the log‐transformed remission status (Table S1). Therefore, we stratified the post‐treatment FPG and PPG levels in the subsequent logistic regression analysis. Setting the stratified boundary value of post‐treatment FPG at 7.0 mmol/L, according to the Classification and Regression Tree algorithms, improved prediction accuracy compared to the mean value or median value. The post‐treatment PPG was grouped by the median value of 9.0 mmol/L. No multicollinear correlations were found between matrices of variables, indicating that the logistic regression model was valid (Table S2).

### 4.3 Influencing Factors of Long‐Term Euglycemic Remission

All significantly different influencing factors in the training cohort were analyzed by logistic regression, and three parameters were identified as independent prognostic factors: FPG after CSII, MG during CSII, and PPG/FPG ratio at baseline. The odds ratios were 0.06 (95% CI, 0.02–0.25), 0.41 (0.18–0.93), and 6.55 (1.39–30.89), respectively (Table 2).

**TABLE 2:** Logistic regression analysis of influence factors on long‐term drug‐free glycemic remission.

	Odds ratio	95% CI for OR	*p*
Age	0.99	0.94–1.06	0.918
Gender	2.35	0.64–8.76	0.200
BMI at baseline	1.15	0.99–1.34	0.075
PPG/FPG at baseline	6.55	1.39–30.89	0.018*
RDW at baseline	0.90	0.75–1.07	0.236
Euglycemia obtained time	0.81	0.38–1.73	0.577
MG during CSII	0.41	0.18–0.93	0.034*
Mild hypoglycemia during CSII	0.67	0.18–2.50	0.547
FPG (≤ 7.0 mmol/L or not) after CSII	0.06	0.02–0.25	< 0.001*
PPG (group by tertiles) after CSII	0.87	0.44–1.72	0.679
AIR after CSII	1.00	0.99–1.00	0.065
Creatinine after CSII	0.99	0.95–1.03	0.633

Abbreviations: AIR: acute insulin response; BMI: body mass index; FPG: fasting plasma glucose; HDL‐C: high density lipoprotein cholesterol; MG: mean maintaining glucose after normoglycemia; PPG: postprandial plasma glucose; RDW: red cell distribution width; TG: triglyceride.

* *p* < 0.05.

### 4.4 Accuracy of the Logistic Regression Model

**FIGURE 2:**
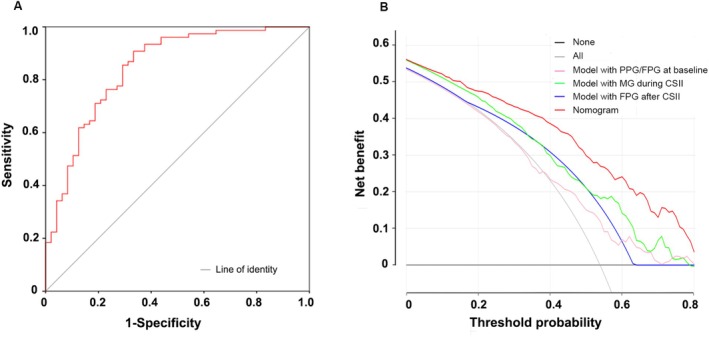
Receiver operating characteristic curve (ROC) of logistic regression model and decision curve analysis (DCA) for the nomogram. (A) The area under the curve (AUC) of the logistic regression model was 0.85 (95% CI, 0.77–0.92), indicating its high level of accuracy and reliability in making predictions. (B) Decision curve analysis (DCA) for the nomogram and logistic model with each one parameter. The *y*‐axis measures the net benefit. The pink line shows the model with PPG/FPG before CSII. The Green one represents the model with MG during CSII. The blue line indicates the model with post‐CSII FPG. The red one demonstrates the nomogram with baseline PPG/FPG, MG during CSII and post‐treatment FPG. The black horizontal line represents the assumption of no patients obtaining the long‐term glucose remission and the gray one means the assumption of all patients attaining glucose remission. When the threshold probability is determined, the nomogram holds the largest net benefit.

A ROC was constructed based on the logistic regression model, and the area under the curve (AUC) of the logistic regression model was 0.85 (95% CI, 0.77–0.92) (Figure 2A). The sensitivity, specificity, positive predictive value, and negative predictive value were 89.47%, 68.75%, 81.93%, and 80.49%, respectively. The overall accuracy was 81.50%, demonstrating good quality in estimating the probability of long‐term glycemic remission.

### 4.5 Nomogram Construction and Validation for the 1‐Year, Drug‐Free Glycemic Remission Probability

**FIGURE 3:**
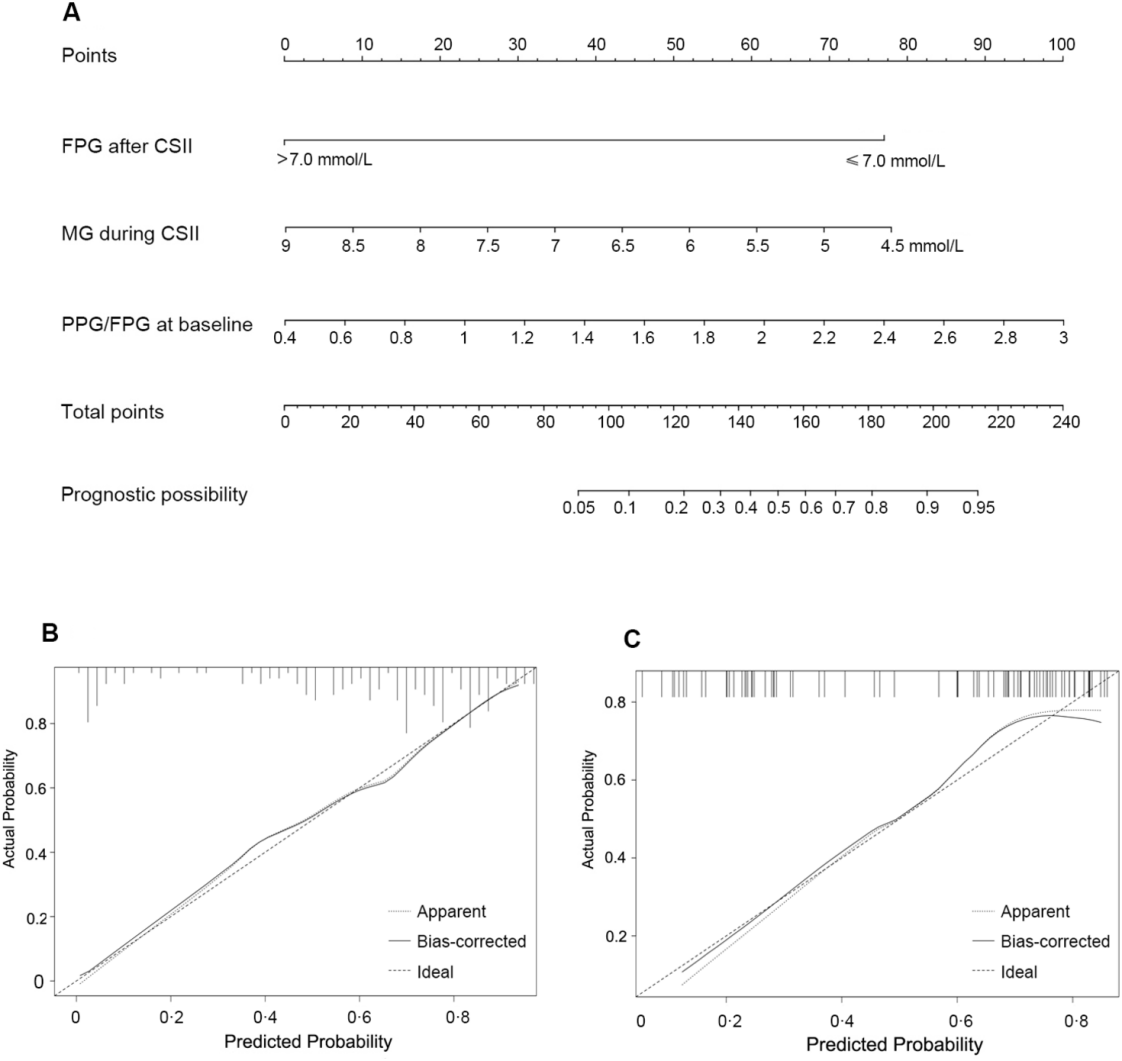
Nomogram for estimation of long‐term glucose remission and its predictive performance. (A) Nomogram is presented to estimate the probability of long‐term drug‐free glucose remission in newly diagnosed type 2 diabetes after 2–3 weeks of intensive insulin therapy. (B) Validity of predictive performance of the nomogram in the training cohort (*n* = 211). (C) Validity of predictive performance of the nomogram in the validation cohort (*n* = 91). Figure (B) and (C) demonstrate good validities and stabilities of the nomogram. The gray and black solid lines represent the performance of the nomogram, of which a closer fit to the ideal line represents a better prediction.

As results showed that the FPG after CSII, MG during CSII, and PPG/FPG ratio at baseline were significant predictors, these factors were combined into an estimation nomogram in the training cohort using R software. DCA was used to compare the benefit between the nomogram and other logistic models containing the separate influencing factors. The results showed that using the nomogram to predict the sustained glycemic remission provided a higher net benefit than using the single influence factor in clinical practice (Figure 2B). The nomogram was shown in Figure 3A. The model was internally validated using the bootstrap validation method. By incorporating these three factors, the nomogram achieved good concordance indexes of 0.81 (0.74–0.88) in the training cohort and 0.80 (0.72–0.90) in validation one and presented well‐fitted calibration curves. The *p* value was > 0.05 in the Hosmer‐Lemeshow test, reflecting a good fit of the model (Figure 3B,C).

### 4.6 Remission Rates in Different Subgroups and With Varying Predicted Probabilities

**FIGURE 4:**
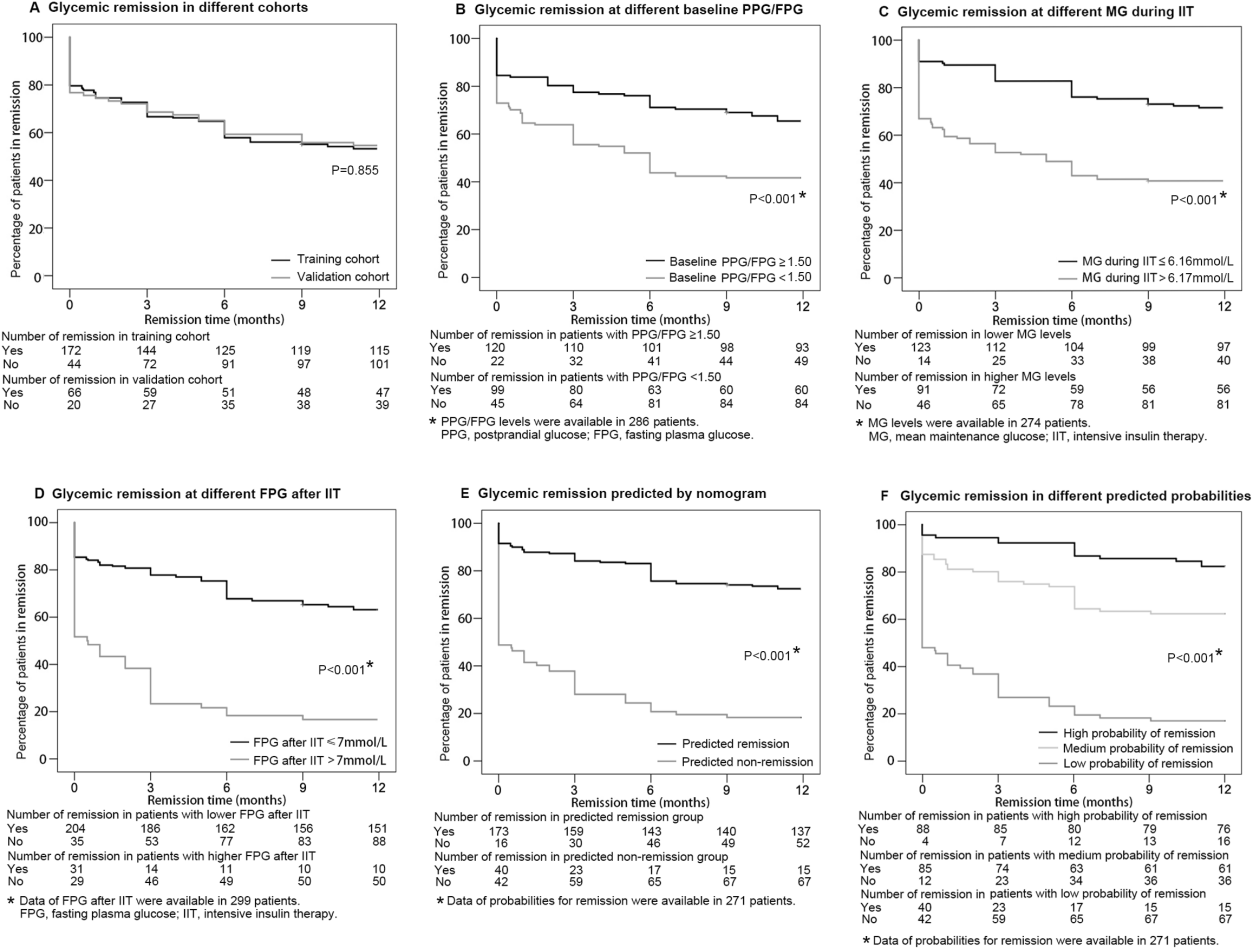
Kaplan–Meier survival curves stratified by the independent influence factors and different nomogram predictions. (A) The remission rates of patients in the training and validation cohorts were similar. (B) The remission rates were different in patients with different PPG/FPG levels at baseline. (C) The remission rate of patients with lower MG levels during short‐term IIT was higher than those with higher MG. (D) Patients with lower FPG levels after the suspension of short‐term IIT had a higher remission rate. (E) The nomogram could well differentiate between patients who would attain sustained remission or not. (F) The prediction of remission probability by nomogram was highly consistent with the actual remission rate. Patients with a predicted remission probability exceeding 0.71 demonstrated a fairly high remission rate, while those with a predicted probability below 0.43 had a rather low remission rate.

The remission rate of patients in the training and validation cohorts at each time‐point was demonstrated in Figure 4A. The specific percentage of remission stratified by the independent influence factors during the one‐year follow‐up was shown in Figure 4B–D.

Determined as the value with the highest Youden index score, the cut‐off value of probability was 0.43 (corresponding total points of 147 in nomogram). Patients predicted with a possibility of remission higher than 0.43 by the nomogram were likely to have a sustained remission compared to those who were not (Figure 4E). Additionally, we conducted a stratified analysis based on the tertiles of the remission possibility and found that patients with a predicted remission probability exceeding 0.71 demonstrated a fairly high remission rate, while those with a predicted probability below 0.43 had a rather low remission rate (Figure 4F).

### 4.7 Clinical Use

An example is presented to demonstrate the application scenario of this predictive nomogram. Suppose a patient has a baseline FPG of 10.0 mmol/L and PPG of 20.0 mmol/L. During the short‐term IIT, the patient's glucose levels are well controlled and the MG is 6.3 mmol/L. After discontinuing insulin therapy for at least 12 h, the post‐treatment FPG is 6.7 mmol/L. How likely would this patient sustain a long‐term glycemic remission without any hypoglycemic medications?

According to the nomogram in Figure 3A, the patient's PPG/FPG value is 2.0; therefore, the corresponding PPG/FPG score is 62 points. The MG is 6.3 mmol/L (47 points), and the FPG after discontinuing insulin therapy is 6.7 mmol/L (≤ 7.00 mmol/L, 77 points). The patient's total score would be 186 (the sum of 62, 47 and 77) points. Thus, the probability of the patient's one‐year glycemic remission will be 83%. As a result, this patient has a good chance of sustained glycemic remission after short‐term intensive insulin therapy.

## 5 Discussion

This study implies that IIT is quite a promising way to mitigate hyperglycemia toxicity, alleviate β‐cell dysfunction, and induce euglycemic remission in patients with newly diagnosed T2DM, even in those at very high baseline glucose levels. Given that the “legacy” effect exists, conferred by earlier improved glycemic control with more beneficial effects on mortality and cardiovascular disease risks over time, guidelines have been influenced to advocate early more intensive glucose‐lowering therapy, such as dual combination therapies of vildagliptin plus metformin in VERIFY (Vildagliptin Efficacy in combination with metfoRmIn For earlY treatment of type 2 diabetes), than the traditional stepwise add‐on strategy [28, 29, 30]. Most patients, however, still do not reach their glycemic targets [31]. Our previous study has demonstrated that IIT has a much better effect than the initial treatment of one or dual hypoglycemic drugs [9]. Nevertheless, nearly half of the patients did not achieve glycemic remission after CSII. In this study, we developed a concise nomogram to predict sustained drug‐free remission. According to the DCA results and Kaplan–Meier estimates, the prediction accuracy of the nomogram is much higher than any single indicator that we have reported before. It provides a quantitative method for predicting long‐term glycemic remission and serves as a reliable basis for making individualized treatment plans. The higher possibility predicted by the nomogram, the greater remission rate is observed.

The overall prevalence of diabetes was estimated to be as high as 12.8% in Chinese adults, yet only 39.7% of those treated had adequate glycemic control [32, 33, 34, 35]. According to the Observational Registry of Basal Insulin Treatment (ORBIT) study, nearly half of the patients treated with hypoglycemic regimens did not report dose titrations during the follow‐up [36]. The “treatment inertia” is prevailing, so that the use of this nomogram would not only prevent inadequate treatment after IIT, which may lead to poor glucose control, but also avoid excessive treatment and waste of medical resources in clinical practice. For patients who are predicted to be able to achieve long‐term, drug‐free glucose remission, only diet and exercise would be recommended. For patients with a predicted probability below 0.43, they are unlikely to achieve remission and, as a result, should undergo more frequent blood glucose monitoring and take hypoglycemic drugs as needed to maintain stable glucose controls [37, 38].

In our previous and ongoing national multi‐center studies, we found that patients in some medical centers had lower glycemic remission rates. In addition to patients' characteristics, it might be mainly due to higher MG during CSII and unsatisfactory FPG after therapy. As indicated by the nomogram, the level of average glucose during CSII treatment is the only factor that can be manipulated. Although guidelines set a target of 4.4–7.2 mmol/L for pre‐prandial glucose and less than 10 mmol/L for the peak value of postprandial glucose, for patients with early T2DM and without severe complications, chances of remission would greatly increase if their glucose levels were more strictly controlled during CSII therapy [10]. Then how to improve glycemic control during CSII? Referring to our previous studies, we have developed a formula for the initial dose of CSII. By using the insulin‐dosing formula, glucose can be controlled faster and more steadily [24]. In addition, patients' attitudes also affect glycemic control during and long after CSII. Therefore, diabetes education and self‐management support should be provided throughout the whole process to improve patients' compliance and glucose control [12, 39, 40].

The present study has several strengths. First, it provides a more accurate visualized prediction method that clinicians could use to easily and promptly calculate individualized remission probability for each patient with newly diagnosed T2DM receiving CSII. Predicting long‐term glucose remission probability may avoid not only insufficient follow‐up treatment and unstable glucose, but also over‐treatment and waste of medical resources. Second, the results emphasize the importance of strict glucose control and complete elimination of glucose toxicity during the short‐term IIT. Remission is less likely if glucose is not well controlled during the IIT. Third, all elements of this nomogram are composed of glucose indexes before, during, and after short‐term insulin treatment, thus reducing the needs for complicated and tedious detection of multiple indexes and facilitating the clinical application.

Although our study, for the first time, to our knowledge, provides a quantitative method for predicting sustained remission in patients with newly diagnosed T2DM after short‐term IIT, there are some limitations. First, the nomogram is only applicable to newly diagnosed T2DM patients who receive short‐term CSII and may not be suitable for other treatment groups. Second, known variables such as β‐cell function and insulin resistance index measured by glucose clamp techniques are not included because of the lack of data, and there may be other markers not yet identified that may be used to predict remission outcomes. Third, it should be noted that the current study is in a single ethnic group, so its generalizability beyond the ethnic group requires further validation.

In summary, short‐term intensive insulin therapy is an effective way to maintain sustained euglycemic remission in patients with newly diagnosed type 2 diabetes, even in those at very high baseline glucose levels. A strict control of glucose during insulin therapy is the sole factor of glycemic remission that can be manually modifiable. We construct a nomogram to quantitatively estimate the probabilities of the one‐year glycemic remission, and thus help to reduce subsequent over‐treatment or clinical inertia after transient intensive insulin therapy.

## Author Contributions

Professor Y.L. designed, organized, and sponsored the series of studies. L.X. and Z.X. analyzed the data and wrote the manuscript under the direction of Y.L. L.X., L.L., and Y.L. verified the underlying data. L.L., Z.H., H.L., J.L., X.H., and W.D. contributed to data collection. Z.X. and Y.L. reviewed and modified the manuscript. Y.L. is the guarantor of this work, who has full access to all data in the study.

## Consent

The authors have nothing to report.

## Conflicts of Interest

Professor Y.L. has unrestricted research funds from Novartis (China) and Medtronic (Shanghai). All other authors declare no conflicts of interest.

## Supporting information


**Data S1:** Supporting Information.
