# Supplementary material for: Construction and Implications of Nomogram for Predicting Sustained Glycemic Remission After Short‐Term Intensive Insulin Therapy in Newly Diagnosed Type 2 Diabetes
Source: J Diabetes. 2025 Aug 1;17(8):e70135. doi: 10.1111/1753-0407.70135 (PMC12314641; doi:10.1111/1753-0407.70135)
Supplement: Supplementary file 1 — Data S1: Supporting Information. [file JDB-17-e70135-s001.docx]

**Supplementary Table 1. The Box-Tidwell method to test the linear relationship between the logit conversion value of influence factors and the remission probability.**

|  | Regression coefficient | P value |
| --- | --- | --- |
| Gender | -0.140 | 0.857 |
| Age | -0.197 | 0.848 |
| BMI | 4.425 | 0.446 |
| PPG/FPG at baseline | -3.950 | 0.645 |
| RDW at baseline | 1.190 | 0.915 |
| Time of achieving euglycemia | -0.383 | 0.679 |
| MG during CSII | 16.873 | 0.289 |
| Hypoglycemia during CSII | 0.531 | 0.489 |
| FPG after CSII | 24.221 | 0.010 ^*^ |
| PPG after CSII | -6.131 | 0.019 ^*^ |
| AIR after CSII | -0.004 | 0.485 |
| Creatinine after CSII | 1.644 | 0.100 |
| Age by ln (age) | 0.036 | 0.865 |
| BMI by ln (BMI) | -1.004 | 0.461 |
| PPG/FPG by ln (PPG/FPG) | 4.350 | 0.458 |
| RDW by ln (RDW) | -0.302 | 0.899 |
| MG by ln (MG) | -6.131 | 0.263 |
| FPG by ln (FPG) | -8.816 | 0.008 ^*^ |
| PPG by ln (PPG) | 2.048 | 0.022 ^*^ |
| AIR by ln (AIR) | 0.001 | 0.535 |
| Creatinine by ln (Creatinine) | -0.310 | 0.103 |

^*^ P<0.05. ln: natural logarithm; BMI: body mass index; FPG: fasting plasma glucose; PPG: postprandial plasma glucose; RDW: red cell distribution width; MG: mean maintaining level after normoglycemia; AIR: acute insulin response.

**Supplementary Table 2. The multi-collinearity test of the variables in logistic regression model.**

|  | Tolerance Value (TOL) | Variance Inflation Factor (VIF) |
| --- | --- | --- |
| Gender | 0.535 | 1.871 |
| Age | 0.631 | 1.585 |
| BMI at baseline | 0.826 | 1.211 |
| PPG/FPG at baseline | 0.825 | 1.212 |
| RDW at baseline | 0.933 | 1.072 |
| Time of achieving euglycemia | 0.585 | 1.710 |
| MG during CSII | 0.413 | 2.421 |
| Mild hypoglycemia during CSII | 0.588 | 1.701 |
| FPG after CSII | 0.617 | 1.622 |
| PPG after CSII | 0.631 | 1.584 |
| AIR after CSII | 0.620 | 1.612 |
| Creatinine after CSII | 0.569 | 1.757 |

BMI: body mass index; FPG: fasting plasma glucose; PPG: postprandial plasma glucose; RDW: red cell distribution width; MG: mean maintaining level after normoglycemia; AIR: acute insulin response.
